# Capacity Building, Knowledge Enhancement, and Consultative Processes for Development of a Digital Tool (Ni-kshay SETU) to Support the Management of Patients with Tuberculosis: Exploratory Qualitative Study

**DOI:** 10.2196/45400

**Published:** 2023-06-19

**Authors:** Harsh Shah, Jay Patel, Sandul Yasobant, Deepak Saxena, Somen Saha, Anish Sinha, Priya Bhavsar, Yogesh Patel, Bhavesh Modi, Pankaj Nimavat, Dixit Kapadiya, Manish Fancy

**Affiliations:** 1 Department of Public Health Science Indian Institute of Public Health Gandhinagar Gandhinagar India; 2 School of Epidemiology and Public Health Jawaharlal Nehru Medical College Datta Meghe Institute of Medical Sciences Wardha India; 3 Department of Tuberculosis Project World Health Partners Noida India; 4 Department of Community & Family Medicine All-India Institute of Medical Sciences Rajkot, Gujarat India; 5 State Training and Demonstration Center, State Tuberculosis Cell Department of Health and Family Welfare Government of Gujarat Ahmedabad India; 6 Office of Regional Deputy Director Department of Health and Family Welfare Government of Gujarat Bhavnagar India

**Keywords:** capacity building, Ni-kshay SETU, National Tuberculosis Elimination Program, digital health, India, tuberculosis

## Abstract

**Background:**

Achieving the target for eliminating tuberculosis (TB) in India by 2025, 5 years ahead of the global target, critically depends on strengthening the capacity of human resources as one of the key components of the health system. Due to the rapid updates of standards and protocols, the human resources for TB health care suffer from a lack of understanding of recent updates and acquiring necessary knowledge.

**Objective:**

Despite an increasing focus on the digital revolution in health care, there is no such platform available to deliver the key updates in national TB control programs with easy access. Thus, the aim of this study was to explore the development and evolution of a mobile health tool for capacity building of the Indian health system’s workforce to better manage patients with TB.

**Methods:**

This study involved two phases. The first phase was based on a qualitative investigation, including personal interviews to understand the basic requirements of staff working in the management of patients with TB, followed by participatory consultative meetings with stakeholders to validate and develop the content for the mobile health app. Qualitative information was collected from the Purbi Singhbhum and Ranchi districts of Jharkhand and Gandhinagar, and from the Surat districts of Gujarat State. In the second phase, a participatory design process was undertaken as part of the content creation and validation exercises.

**Results:**

The first phase collected information from 126 health care staff, with a mean age of 38.4 (SD 8.9) years and average work experience of 8.9 years. The assessment revealed that more than two-thirds of participants needed further training and lacked knowledge of the most current updates to TB program guidelines. The consultative process determined the need for a digital solution in easily accessible formats and ready reckoner content to deliver practical solutions to address operational issues for implementation of the program. Ultimately, the digital platform named Ni-kshay SETU (Support to End Tuberculosis) was developed to support the knowledge enhancement of health care workers.

**Conclusions:**

The development of staff capacity is vital to the success or failure of any program or intervention. Having up-to-date information provides confidence to health care staff when interacting with patients in the community and aids in making quick judgments when handling case scenarios. Ni-kshay SETU represents a novel digital capacity-building platform for enhancing human resource skills in achieving the goal of TB elimination.

## Introduction

Tuberculosis (TB), caused by *Mycobacterium tuberculosis*, is a significant public health concern in developing countries such as India. Despite being a preventable and curable disease, TB kills 1.4 million people a year worldwide, making it the leading global infectious disease killer and a major contributor to antimicrobial resistance [1]. In 2021, 10.6 million people became infected with TB globally, and India has the highest TB incidence worldwide, with an estimated number of 2.1 million cases in 2021 [1,2]. To combat this massive disease burden, a comprehensive, practical, and long-term extended strategy is required across the nation. Accordingly, India has planned to achieve TB elimination by 2025, 5 years ahead of the global agenda [3], under its flagship National TB Elimination Program (NTEP).

Health care workers under the NTEP are critical in reducing the burden of TB-related problems. Despite being engaged and trained in the program, the ever-changing program guidelines, introduction of new strategies and innovations, and continuous changes in the mode of care services delivery within the health system pose challenges in keeping the entire workforce up to date with all necessary information [4]. Health care workers must possess recently updated knowledge to improve TB notification and/or the status of TB-free disease. Among other challenges, lack of guidance on their engagement, the flow of information for NTEP updates, and the type of involvement in each cascade of TB care remain unclear. This problem is more significant in two specific workforces: the general health care workers who are indirectly engaged in TB elimination and the new joiners of the NTEP. Even though every health care program is required to continuously build capacity within its vertical structure, collaborating with the general health system and vertical programs such as the NTEP to build capacity has proven to be the most difficult task.

Although continuous refresher training helps those working in the field strengthen their knowledge, develop a positive attitude, and prevent the spread of TB in the community, the COVID-19 pandemic added a significant challenge to physical capacity building and direct support/training [5,6]. Although digital technology platforms are not new, COVID-19 has served as a strong motivator to the widespread adoption of available digital solutions for disease prevention and control.

Digital technology holds a promising future in dealing with capacity-building issues among health care workers [7]. In particular, this technology enables designing platforms according to a specific need for an intervention in the field, which can ultimately improve knowledge and provide a customized solution to address any questions. Digital solutions can also help to close knowledge gaps, provide the best possible patient care, and implement proper infection control measures [8-10]. Moreover, digital technology can serve as a platform to provide necessary health education for interventions and to assess the need for training in specific areas where health care providers’ knowledge can be strengthened [11,12]. Such tools can also empower service providers to manage patients’ health more effectively and efficiently by providing primary care from the field. Overall, this technology can reduce the delay in transferring knowledge from the state to facility and frontline worker levels [13,14]. Despite the development of many digital health platforms in TB care, such as Ni-kshay [15], TB Aarogya Sathi [16], Swasth e-Gurukul [17], and the World Health Organization (WHO) TB Guide [18], there is a lack of evidence on digital capacity building of the health workforce for TB.

Thus, the aim of this study was to showcase the evolution of the design of a digital platform for the capacity building of Indian health systems in TB care. Further, this study provides a skeleton of digital health innovations and their process, describing the design and delivery of a new digital platform as a ready reckoner and decision-making tool for the health workforce to better manage TB.

## Methods

### Design

The study was performed using a two-phase approach from March 2021 to January 2022. First, a qualitative research methodology was used to understand the key gaps and the need to enhance knowledge among health care workers working directly or indirectly with the NTEP. The synthesis of data collected from this first phase initiated the second phase to address the need to strengthen the capacity-building component through development of a digital health solution for sustainable implementation of the NTEP.

### Study Setting

#### Phase One: Qualitative Research

The qualitative research phase of the study was performed in the Purbi Singhbhum and Ranchi districts of Jharkhand state and in the Gandhinagar and Surat districts of Gujarat state. Under the NTEP, a district is divided into TB Units (TUs), each covering an approximate population of 0.5 million. A total of 13 TUs were identified as an area for this qualitative investigation. Each TU has a Designated Microscopy Center (DMC), each covering a population of approximately 0.1 to 0.12 million. Under the NTEP’s integration policy, each DMC is located on the government’s general health facility premises. A DMC represents the crucial entry point or “gateway” for a patient with suspected or confirmed TB to access the TB diagnostic and treatment services offered by the NTEP. Any patient with suspected or confirmed TB must officially visit the DMC to enroll in the TB program. In addition, other government-run and private health facilities are operating in the areas surrounding the DMCs.

#### Phase Two: Consultation

Various stakeholders from the state (Gujarat and Jharkhand) and central health divisions were consulted on the design process of the digital platform.

### Study Sampling and Process

#### Phase One: Qualitative Research

**Figure 1:**
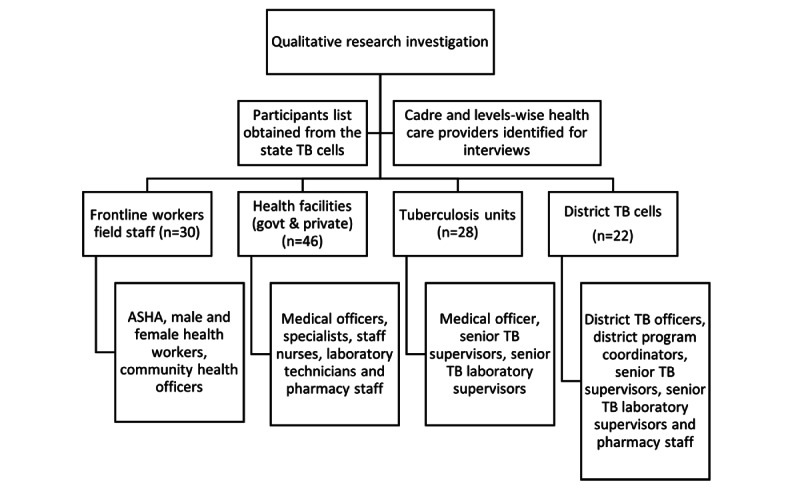
Flow chart on the sample selection for the qualitative research investigation (N=126). ASHA: accredited social health activist; govt: government; TB: tuberculosis.

A purposive sampling strategy was applied to ensure diversity of cadres and maximum variation in responses for interviews, resulting in recruitment of 126 health care staff directly or indirectly involved in TB patient care. The line lists of the staff from the study settings were obtained from the state TB cell offices. Cadre-wise and work-wise distributions were applied to the line lists at the levels of health facility, TU (block), district, and state. The study participants were from the public sector (ie, central and state TB cells, district TB centers, TB units, and health facilities) or consultants from various organizations and private settings (especially doctors and their assistants). Additionally, field-level health staff such as community health officers, multipurpose workers, and accredited social health activists (ASHAs) were included in the study (Figure 1).

#### Phase Two: Reflection on Qualitative Findings and Design Process for a Digital Solution

The second phase involved a consultative group for a participatory discussion or one-to-one meetings with NTEP stakeholders to understand their views on the requirements for capacity building of health staff. The consultative meetings were not structured with a defined methodology and the stakeholder consultations aimed toward developing solutions that address the knowledge gaps and needs of the health care workers. There were 46 consultative meetings conducted nationwide with multiple stages to seek the reflections of stakeholders on the qualitative study findings from phase one and to consult them on the development of the digital solution. The participants were from the government (state and central levels), technical experts from private organizations and medical colleges, along with clinical specialists from the pulmonology field.

### Data Collection and Synthesis

#### Phase One: Qualitative Research

In-depth one-to-one interviews were conducted in the regional language using an interview guide developed by the principal investigator and coinvestigators trained in NTEP guidelines. Consent was obtained and interviews were electronically recorded. The interview guides were pilot-tested and questions were restructured as needed. Social cognitive theory was used as a guiding framework for this study as it helps to understand human actions, intuitions, motivations, and processes of behavior change that determine training needs. There were 126 study participants with their assigned days’ availability to provide open-ended qualitative interviews. The interviews were continued until saturation of the responses from the participants was reached. After each interview, topics-based memos were written that enabled capturing different perspectives to address and create solutions based on the training need requirements of health staff for strong implementation of the NTEP. In some cases, participants were revisited to gather more insight.

Audio-recorded data from interviews of health care providers were transcribed verbatim and translated into English. During the interview, verbatim notes were taken. On the same day of the interviews, transcripts were created using the verbatim notes of a coinvestigator in the field. The principal investigator read the collected transcripts to become familiar with the data once compiled. The transcripts were analyzed manually using descriptive content analysis. A second researcher skilled in qualitative research techniques reviewed the data to check for credibility and eliminate bias. The coding conventions and the theme creation were determined by consensus and according to accepted practices [19]. Any differences were resolved by discussion. This phase lasted for the 4 months of the total study period (March 2021 to June 2021).

#### Phase Two: Reflection on Qualitative Findings and Design Process for a Digital Solution

**Figure 2:**
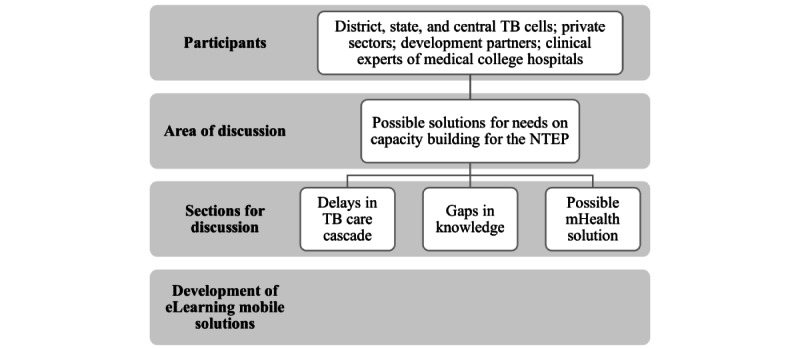
Flow diagram for determining the needs of health care providers in management of patients with tuberculosis (TB). mHealth: mobile health; NTEP: National Tuberculosis Elimination Program.

The second phase included group or individual participatory discussions with stakeholders about outcomes related to the themes and findings from the first phase of the study. This consultative phase was designed to address the needs of health care workers in the management of patients with TB in line with national NTEP guidelines. The end goal of the discussions was to determine potential solutions to solve knowledge gaps experienced by health care workers. There were three areas of focus to these discussions: (1) delay in services to patients with TB, (2) gaps in knowledge of health care providers that lead to delays in services, and (3) possible digital solutions to enhance the knowledge of health care providers (Figure 2). An iterative discussion process was applied to achieve consensus and reach a final solution to the entire exercise. The consultation and building of a digital solution was undertaken from June 2021 to January 2022.

### Ethical Approval

Approval for carrying out this study was obtained from the Institutional Review Board of the Indian Institute of Public Health, Gandhinagar, Gujarat (TRC-IEC 18/2020-21). Permission for carrying out the study was obtained from the State TB Office, Government of Gujarat and Jharkhand State. Written consent was obtained from all study participants.

## Results

### Digital Platform

The qualitative enquiry and consultation exercise culminated in development of a digital platform named Ni-kshay SETU (Support to End Tuberculosis). This digital platform [20] is available free of cost to health care workers involved in TB care. Further, both Android [21] and iOS [22] versions are available in the respective app stores. Below, we describe the detailed evolution process for development of Ni-kshay SETU during the two phases of enquiry and the associated findings.

### Phase One: Qualitative Interviews

#### Participant Characteristics and Main Themes

The participants from different health system levels were interviewed during the study’s first phase. The study participants comprised 30 frontline workers from the field, 46 staff members from government and private health facilities, 28 from the district level, and 22 from state-level cadres.

The mean age of the 126 participants was 38.4 (SD 8.9) years and the average work experience was 8.9 years (median 7, IQR 5-20 years), with the maximum level of experience at the district and state levels. The district and state program functionaries are mostly supervisory cadres who are promoted from the cadres of lower levels. Approximately half of the respondents were women, one-third of whom had completed higher education with a bachelor’s degree or above.

Approximately 83% of the health workers indicated the need for refresher trainings. The themes generated from the analysis of the transcripts of in-depth interviews are shown in Table 1.

**Table 1:** Common themes, challenges, and possible solutions that emerged during in-depth interviews with study participants (N=126).

Themes	Challenges	Possible solutions
Knowledge gaps lead to delays in care during the TB^a^ care cascade	Irregular and inadequate trainings of health care workers, including induction training, refresher training, training calendar, private sector staff, and cadre-specific training	Creation of a training calendar and cascade, specifically designed training materials (cadre-based), creation of a training mode
Need for a decision-making support tool on interventions across the TB care cascade for management of patients with TB	Poor management of patients with TB due to complex protocols and algorithms for extrapulmonary, pediatric, and drug-resistant TB; prolonged period of treatment; loss of follow-up or treatment failure of patients	Easy access of reference material for TB patient screening, diagnosis, and treatment; easy understanding of the protocol and algorithms; operational solutions for management of TB patients during day-to-day work; prevention of unwanted outcomes
Communication on newer updates	Communication gaps at each level for sharing latest guideline updates; no system of knowledge assessment	Communication channel for all TB-related updates, including a digital platform and periodic knowledge assessment
Availability of easy-to-read content on a specific section of different NTEP^b^ guidelines	Lengthy and difficult to remember guidelines and protocols; nonavailability of resource materials in a central location	Easy navigation and easy-to-read content, including one package in ready reckoner form and a digital repository in one place with audio-visual resource material
Financial limitations in the training budget	Limited budget, infrastructure, and numbers of trainers	Sufficient financing, infrastructure and workforce, and technical capacity

^a^TB: tuberculosis.

^b^NTEP: National Tuberculosis Elimination Program.

#### Theme 1: Knowledge Gaps Lead to Delays in Care During the TB Care Cascade

The need for knowledge on the management of patients with TB was not assessed at the time of the program implementation plan. Since many new updates and the last formal training took place a few years ago, each cadre of the NTEP staff urged refresher training. A few frontline and health care facility staff were recently appointed without orientation or training. There was an ultimate need to create a planned training calendar based on the needs assessment.

Refresher training is important. New things are updated in the program. If the regimen changed and we are not aware, then we administer the old regimen only, so refresher training is required.

Field worker, TB health visitor with 2 years of experience

Modules for all cadres need to be developed; currently, modules of frontline workers do not contain newer updates on diagnostics and regimen. The delay in universal drug susceptibility testing leads to poor management of TB patients.

Multipurpose health worker, Ranchi, Jharkhand

I am newly joined here and do not know how to manage a drug-resistant TB patient. Also, whenever extrapulmonary TB patients come, I refer them to a higher center due to lack of knowledge on extrapulmonary TB patients.

Medical officer, peripheral health institution, Gandhinagar, Gujarat

#### Theme 2: Need for Refresher Training and a Digital Solution on Interventions Across the TB Care Cascade for the Management of Patients With TB

For each stage of the TB care cascade, the NTEP has several interventions with specific protocols for providing care. Although health institutions have paper copies of these guidelines, a digital solution with easy navigation and a ready reckoner format would help medical personnel to take proper actions when dealing with patients with specific problems.

Training needed for different diagnostic algorithms like pulmonary TB, extrapulmonary TB, pediatric TB, drug-resistant TB, and programmatic management of drug-resistant TB patients is required for medical officers. Refresher training for all health staff specific for programmatic management of drug-resistant TB guidelines on every yearly basis is required due to frequent changes in drug regimens.

Medical officer, health facility, Gujarat

Many patients were not put on the right regimen due to knowledge gaps in diagnostic algorithms, and the delay happened in the treatment care cascade. Decision-making content and cross-checking of knowledge in the form of periodic assessment can be helpful.

Private pulmonologist, Jharkhand

#### Theme 3: Communication on Newer Updates of NTEP Interventions

The NTEP has undergone many newer updates since implementation of the National Strategic Plan 2017-25, with the introduction of injection-free regimens and newer drugs such as bedaquiline and delamanid, along with newer guidelines of preventive treatment for TB infection (Programmatic Management of Tuberculosis Preventive Treatment) [23].

We have issued office orders based on communication received from the Central TB Division, Government of India. We issued to the districts and districts issued to blocks and health facilities. Still, many staff do not receive the communication due to their absence on the day of orientation or longer leaves for other personal commitments.

Senior medical officer, State TB Training and Demonstration Center, Gujarat

We call all the staff members at the health facility whenever we receive the office orders on newer guidelines or instruction for any activity, but not all ASHAs, auxiliary nurse midwives, and male health workers remain present due to their area work. During the COVID-19 pandemic, it was difficult to conduct any meetings or sensitize them to newer updates on any program, not just the TB program.

Medical officer, health facility, Jharkhand

#### Theme 4: Availability of Easy-to-Read Content on a Specific Section of Different NTEP Guidelines

The program guidelines, patient management protocols, repository of all government orders, and demand generation resource materials are available on the national website of the NTEP. During the interviews, it was observed that very few of the participants were using the online platform due to the time taken to search. Moreover, health staff had to go through several pages of guidelines to find a specific component they were looking for.

There are so many pages in programmatic management of drug-resistant TB guidelines. If I want to read or refer to any specific sections it is difficult to search between chapters.

Senior treatment supervisor, Jharkhand

It is difficult for me to read all the guidelines as I have joined recently. Very few materials are available in Hindi, and I find it difficult to manage a TB patient without the trainings.

Male health worker from the field, Jharkhand

Most of the time, we get PowerPoint slides or PDF files to read the protocol or guidelines. I find it difficult to go through all the material. A one-stop solution would be helpful with videos also.

Senior TB treatment supervisor, Gujarat

#### Theme 5: Financial Limitations in the Training Budget of the NTEP

Through interviews, we further found that increasing training coverage to meet the needs of health personnel in capacity building is financially constrained. The infrastructure and funding in place would not be sufficient to train the entire workforce annually.

We have limited resources in terms of infrastructure and budget to cover all staff for 5-7 days of modular training of NTEP. We also need finance to create the state-specific resource material in vernacular language and training materials to give to the trainees.

District TB officer, Gujarat

We are not training private sector staff. We do not have that much money in the budget. We orient the doctors, and it is their responsibility to train them, but we provide resource materials in soft copies.

Block medical officer, Gujarat

### Phase 2: Consultative Discussions with Stakeholders and Creation of a Digital Health App

#### Main Discussion Points and Proposed Solution

**Figure 3:**
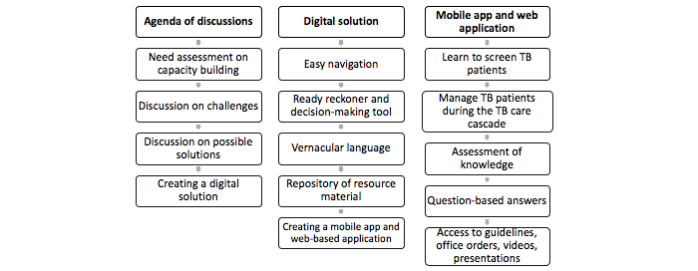
Discussion points with stakeholders for the creation of a digital solution. TB: tuberculosis.

This phase’s consultative procedure included a series of formal and informal discussions with various stakeholders, including frontline workers. The early discussion exercises resulted in agreement on the need for a digital tool in the shape of a digital health app that boosts the capability of health cadres and supports and helps them with TB diagnosis and treatment concerns. To develop the digital solutions, different areas were discussed so as to build a prototype and then test it with health care providers. The end result was the development of Ni-kshay SETU that provides easy access to the most recent guidelines in the local languages of Gujarati, Hindi, and English, as well as practical answers to operational questions that health care workers frequently encounter in their day-to-day work (Figure 3).

#### Content Creation and Pilot Testing for Understanding the User Experience

Initially, a ready reckoner version of evidence-based content from current NTEP guidelines was incorporated into the digital modules, and its compliance with the guidelines was verified by experts. The landscaping of available knowledge and TB control program apps such as Ni-kshay [15], TB Aarogya Sathi [16], Swath e-Gurukul [17], N-TB [24], and WHO TB Guide [18] were reviewed. To ensure context relevance, health care workers provided feedback on the appropriateness of the user interface, sections of the interface, technical content, and an early prototype of the digital app. The iterative process used during the development of the digital app is shown in Figure 3. The challenges and proposed solutions (Table 1) were addressed by designing a user-responsive interface and the cadre-specific content in the form of a digital app (Textbox 1).

From October to December 2021, a beta version of the app was shared for input on user interface design and to identify key app components. Based on the feedback, the content of the app was evaluated and prioritized. The design and content of the app were put to the test by a variety of cadres, including field staff, medical officers at peripheral health institutions, community health officers, senior treatment supervisors, senior TB laboratory supervisors, private doctors, and general health care personnel.

**Figure 4:**
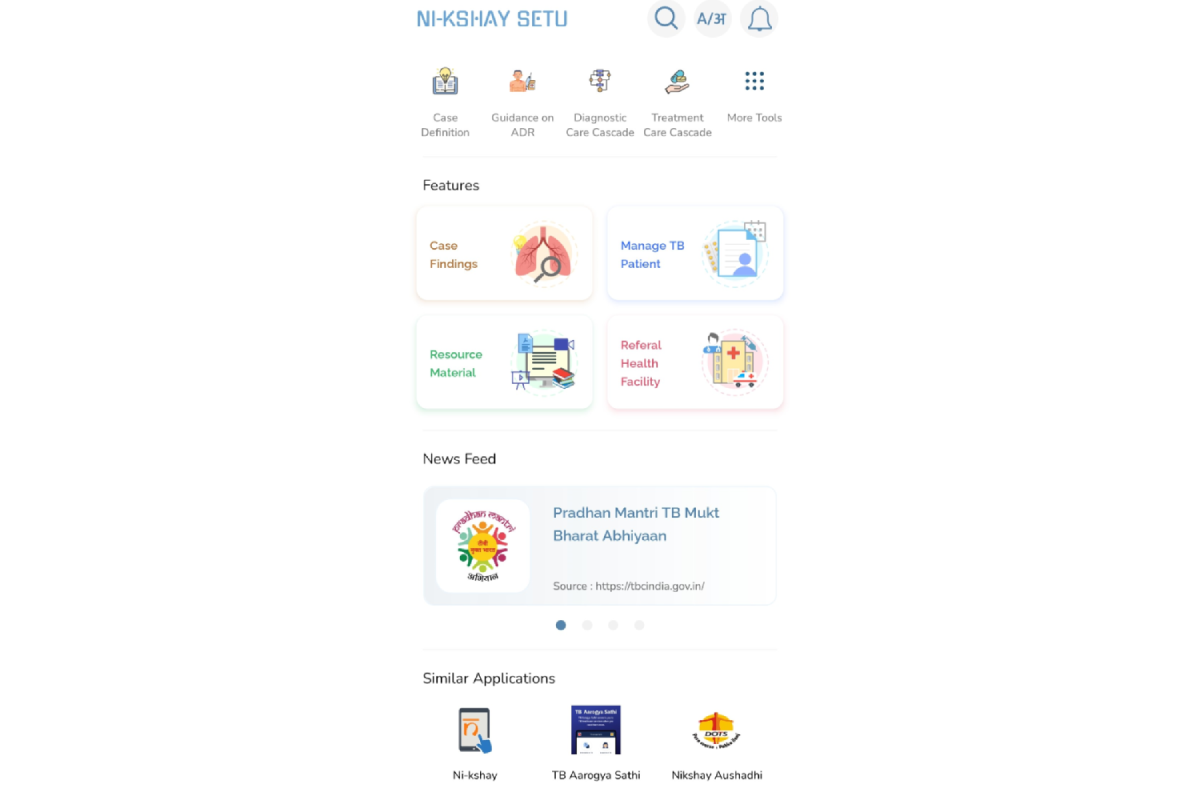
Mobile app interface.

Finally, the Ni-kshay SETU app was developed for web, iOS, and Android platforms focusing on the core components of the management of patients with TB during the care cascade. The core components are how to identify a patient with TB, how to manage the patient, and where to refer a patient, along with resource materials and assessment sections. The app interface is shown in Figure 4. The Ni-kshay SETU app has been rolled out in 35 states and union territories in India to date, with more than 18,400 subscribers on board and 540,000 visits of the app.

**Key features of the Ni-kshay SETU digital app based on consultations with stakeholders to address the challenges and solutions raised in interviews (see Table 1).**

- Digital learning platform as a mobile app
- Patient-centric diagnostic and treatment algorithm to guide users for management of patients with tuberculosis (TB)
- Cadre-specific modules in the form of ready reckoner content with easy navigation
- Access to a digital repository with audio-visual resource materials; option to upload state-specific resource materials in local languages
- Artificial intelligence and voice-assisted chatbot in providing specific answers in user-targeted query options
- Assessment sections for all health cadres and specific topics of patient management
- Backend analytical dashboard to monitor the usage of the content and to identify training needs
- Multiple Indian language support for health care workers from varied geographical locations

## Discussion

### Principal Findings

The National Strategic Plan 2017-25 was developed to reduce the burden of TB and eliminate the disease by 2025. This plan focuses on four critical strategic pillars: “Detect-Treat-Prevent-Build.” With “Build” as one of the strategic pillars, major attention has been paid to developing the ability of human resources and enhancing the enabling policies [23]. There are various cascade gaps and challenges encountered in treating a patient with TB and achieving a successful outcome, including the difficulty in identifying patients with TB; diagnosing TB, particularly extrapulmonary TB; initiating treatment; managing adverse drug reactions; treatment adherence; and follow-up of the patient after successful treatment completion [25].

This study emphasizes the need for health care staff capacity-building and the availability of an easy-to-access solution to solve the issues they confront when managing patients with TB. The lack of updates on the newly released NTEP guidelines indirectly prolongs delays across the TB care cascades [25]. This gap also impacts health care providers’ judgment process while working per national guidelines and managing patients with TB. Therefore, our study also focused on the fundamental requirements of field employees, allowing them to take rapid and spontaneous action to solve the issues they encounter during NTEP implementation.

Various studies highlighted that mobile apps, or digital health solutions, improve people’s health worldwide [26]. Health care professionals can handle patients with more independence, effectiveness, and confidence owing to the information’s easy readability and availability through digital health apps [27,28]. These digital tools have been demonstrated as a straightforward, dependable, and economical method for enhancing patients’ lung health, physical exercise, and comorbidity management [29-36]. Digital solutions are advantageous and have the potential to address the needs of health care systems, particularly in low- and middle-income countries, which face challenges such as a lack of medical professionals, limited funding, a high disease burden, high morbidity and mortality rates, and challenges in providing health services in rural and tribal areas.

This study focused on the need for health care professionals to have a quick reckoner and decision-support tool that is easily accessible and that enables them to take various actions based on the issues and complexity they run into when implementing the NTEP, even in remote stations. The app was created to meet the requirements of the health care workers to manage patients with TB based on the conclusions and observations made during the qualitative interviews. Health care workers may take swift action and have access to updated content at any time and location with the help of Ni-kshay SETU. Additionally, this app offers material in multiple languages that health care personnel may find useful. Along with a resource library with government office orders, guidelines, presentations on clinical standards, and easy-to-learn videos. Ni-kshay SETU also offers content filtering based on cadre and a selection of chatbot questions to help health care staff with their issues.

This study thus offers a platform for the development of more operation research to address capacity-building issues and determine the optimum approach to include digital health learning apps into current implementation frameworks. More research will be performed to determine the impact of Ni-kshay SETU on the TB care cascade of patients by enhancing the knowledge of health care workers.

### Strengths and Limitations

The aim of this study was to gain understanding of the expectations placed on medical personnel working for the NTEP. The study’s restricted geographic scope makes it difficult to generalize the findings. The health staff highlighted a ready reckoner tool as a core requirement because the program is frequently altered depending on technical inputs and field observations. However, this effort represents the first creation of an app with consideration of the needs of the community personnel working with the TB control program. Therefore, this research serves as a crucial tool for planning and developing the app as well as for building modules and app-related material. Additionally, it is recommended to integrate such an app with multiple platforms to offer health professionals a uniform user experience.

### Conclusions

Health staff represent the primary contact point for a patient with TB. Therefore, it is critical to place our hope on the ability of health care workers to effectively comprehend the technical queries of the program. An easily accessible source of information can direct health care personnel to take the appropriate steps to manage patients with TB at their level. The recently developed Ni-kshay SETU app provides an opportunistic platform to strengthen the health workforce and achieve the target of TB elimination by 2025. The vision of this app is not only to strengthen capacity but also to provide a case for low-cost digital capacity building in resource-constrained settings toward developing a resilient health system.

## Abbreviations

ASHA: accredited social health activist
DMC: Designated Microscopy Center
NTEP: National Tuberculosis Elimination Program
SETU: Support to End Tuberculosis
TB: Tuberculosis
TU: Tuberculosis Unit
WHO: World Health Organization

## Data Availability

All data supporting the conclusions of this study are included within the article.
